# Gut Microbiome-Related Anti-Inflammatory Effects of Aryl Hydrocarbon Receptor Activation on Inflammatory Bowel Disease

**DOI:** 10.3390/ijms25063372

**Published:** 2024-03-16

**Authors:** Salvinaz Islam Moutusy, Seiichiroh Ohsako

**Affiliations:** 1 Laboratory of Environmental Health Sciences, Center for Disease Biology and Integrative Medicine, Graduate School of Medicine, The University of Tokyo, Tokyo 113-0033, Japan; ohsako@m.u-tokyo.ac.jp; 2 Division of Immunology and Rheumatology, Department of Medicine, Stanford University, Stanford, CA 94305, USA; 3 VA Palo Alto Health Care System, Palo Alto, CA 94305, USA

**Keywords:** inflammatory bowel diseases (IBD), aryl hydrocarbon receptor (AHR), gut microbiota, inflammation, dysbiosis

## Abstract

Inflammatory bowel disease (IBD) is one of the most prevalent chronic inflammations of the gastrointestinal tract (GIT). The gut microbial population, the cytokine milieu, the aryl hydrocarbon receptor (AHR) expressed by immune and nonimmune cells and the intrinsic pathway of Th-cell differentiation are implicated in the immunopathology of IBD. AHR activation requires a delicate balance between regulatory and effector T-cells; loss of this balance can cause local gut microbial dysbiosis and intestinal inflammation. Thus, the study of the gut microbiome in association with AHR provides critical insights into IBD pathogenesis and interventions. This review will focus on the recent advancements to form conceptional frameworks on the benefits of AHR activation by commensal gut bacteria in IBD.

## 1. Introduction

Inflammatory bowel disease (IBD) refers to a chronic, remitting, and relapsing inflammation of the gastrointestinal tract (GIT) comprising of two clinical entities, Ulcerative colitis (UC) and Crohn’s disease (CD), which can be differentiated by clinical, histopathological, and endoscopic features [1,2,3]. UC is a chronic mucosal inflammatory process that involves the distal colon leading to ulcerations, severe bleeding, and toxic megacolon, while CD affects the entire digestive system [4]. IBD is characterized by inflammation of the gastrointestinal tract leading to symptoms like changes in bowel habits, stomach pain, and severe weight loss [1]. The exact pathogenesis of IBD is not well understood but it is believed to be more common in genetically predisposed individuals with gut bacterial dysbiosis, disrupted mucosal barrier, and an exacerbated immune response [5,6]. Though current treatment modalities only aim to establish an anti-inflammatory remission rather than any permanent cure, which is burdensome [7], novel treatments are emerging as our understanding of the pathophysiology of IBD expands. The treatment modalities of IBD target a variety of immunological mechanisms, including reduced lymphocyte trafficking to the intestine, blocking and neutralizing pro-inflammatory cytokines, and reducing CD4+ T-cell subset development with anti-inflammatory drugs, immunosuppressive drugs (like azathioprine), antibiotics, and biologic treatments [4]. Biologic agents classified as IBD therapeutics include monoclonal antibodies against tumor necrosis factor-alpha (TNF-α), such as certolizumab, adalimumab, golimumab, and infliximab; agents targeting integrins, such as natalizumab (which targets the α4 subunit in α4β7 and α4β1 integrins, approved for CD); and ustekinumab (a monoclonal antibody directed to the p40 subunit of IL-12 and IL-23). Apart from biologics, small-molecule agents such as anti-JAK and anti-sphingosine 1 phosphate (S1P) medications have gained recognition in the treatment of IBD. Targeting the Janus Kinase Signal Transducer and Activator of Transcription (JAK-STAT) pathway is a potentially effective therapeutic strategy for IBD because cytokine mediators of inflammation, including interferon-gamma (IFN-γ) and interleukin (IL-9, IL-12, and IL-23), rely on the signaling of this pathway and Sphingosine-1-phosphate is a sphingolipid ligand that binds to G protein-coupled receptors (S1P1–S1P5), controlling lymphocyte migration from lymphoid tissue into circulation. JAK inhibitors (tofacitinib, filgotinib, and upadacitinib) and S1P receptor modulators (Ozanimod and Etrasimod) are being investigated for the treatment of IBD. The selection of these agents is influenced by the patient’s co-morbidities, the IBD phenotype and behavior, and the potential side effects of therapy [8]. Although severe conditions still require intestinal resection surgery [9] with more intensive medical care, surgical rates appear to be declining, according to the most recent data from Hungary, the UK, and Canada [10]. Fecal microbiota transplantation (FMT) is a recent treatment for IBD that was evaluated in pediatric IBD patients as well as in healthy family donors [11].

**Figure 1:**
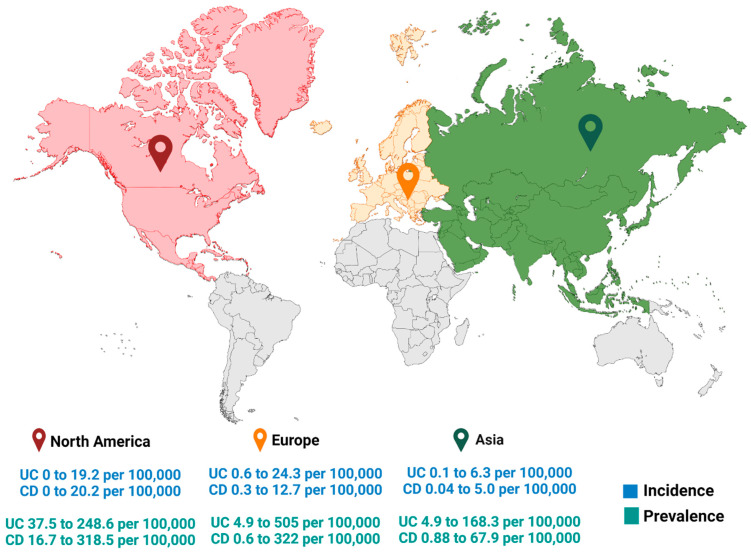
Geographical distribution of highest annual incidence and prevalence rates of Ulcerative colitis (UC) and Crohn’s disease (CD) [13].

IBD is becoming more prevalent worldwide; from 1990 to 2017, the prevalence increased by roughly 6% [12]. A systematic review published in 2010 found that the highest incidence was seen in North America and Europe [13]. An estimated 2.5–3 million people in Europe are thought to have IBD, according to new epidemiological data, which also suggests that the diseases’ incidence and prevalence are rising [14] (Figure 1). IBD occurs in about 0.7% of the total Canadian populations and by 2030, it is expected to affect over 400,000 Canadians [13,15]. Population-based studies on the incidence or prevalence of Crohn’s disease or Ulcerative colitis published in 1990 or later indicate that IBD has become a global health concern in the twenty-first century, with an increase in regions of Africa, Asia, and South America, suggesting that a Westernized lifestyle may be associated with the condition [16]. Moreover, the high cost per patient and the high burden of illness of IBD symptom management and lifestyle modifications are estimated at about USD 2.8 billion annually due to its high prevalence in Canada [17].

The higher incidence of IBD in Westernized countries indicates that IBD results from a complex interaction between multiple etiological factors including genetic susceptibility contributing to the loss of immunological tolerance, the increased availability of autoantigens, and environmental factors such as diet, lifestyle, the use of antibiotics, imbalance of the gut microbial community, disruption of the mucosal barrier resulting in altered immune response, and intestinal inflammation [18,19,20]. Research on the role of microbiome dysbiosis, which refers to the imbalance of gut microbes that eventually becomes detrimental for the host was conducted, though it is still unclear if these changes are the cause or the result of IBD [21]. A symbiotic homeostasis that identifies and eradicates pathogenic bacteria while maintaining homeostasis in the gastrointestinal tract has allowed the intestine and its immune system to evolve, and alteration of microbial communities plays a critical role in the emergence of IBD [22]. Short-term antibiotic therapy in conjunction with anti-inflammatory medications was found to reduce inflammation by altering the gut flora. The gut bacterial translocation may be the source of antigenic stimulation to Toll-like receptor (TLR) ligands to induce this altered immune response [23,24]. While the precise antigens causing an exaggerated immune response in IBD are still unknown, recent advancements in our understanding of the molecular pathogenesis of the disease suggest that gut commensal bacteria are probably the source [25]. It is possible that FMT and other therapies that modify the host–microbiota interactions will lessen the side effects of immunosuppressive and biologic medications [26] but more research is needed to evaluate the long-term safety of FMT. Competing evidence has demonstrated a critical role for the aryl hydrocarbon receptor (AHR) and the gut microbiota in the pathogenesis of IBD. In this review, we discuss the crosstalk of AHR and gut bacterial dysbiosis in IBD from recent advances.

## 2. Role of AHR in IBD

### 2.1. AHR Ligands and the Classical Function of AHR

AHR was initially identified as the receptor for an environmental aromatic hydrocarbon, 2,3,7,8-tetrachlorodibenzo-p-dioxin (TCDD) [27]. AHR is a cytoplasmic ligand activated basic Helix-Loop-Helix-Per-ARNT-Sim transcription factor consisting of the DNA-binding bHLH domain at the N-terminal, the PAS domains (PAS-A to form a complex with ARNT and PAS-B, essential for HSP90 and ligand binding), and the transactivation domain (TAD) at the C-terminal regions [28]. The classical pathway of AHR activation involves the binding of ligands to the receptor regulating the expression of multiple genes. AHR is found in the cytoplasm in an inactivated state, where it is a component of a protein complex with the 90,000 molecular weight heat-shock protein (hsp90) and the c-SRC protein kinase. After binding to a ligand, AHR changes its conformation to move from chaperones and into the nucleus. AHR forms a heterodimer with the nuclear translocator of the aryl hydrocarbon receptor (ARNT) which initiates transcriptional activation from the xenobiotic responsive element (XRE) by binding to the target DNA sequence [28]. Through interactions with other transcription factors, including nuclear factor-kappaB (NFκB), retinoic acid receptor, estrogen receptor (ER), and members of the signal transducers and activators of the transcription family, AHR affects several physiological processes [29]. AHR activation can occasionally suppress NFκB activity, an important inflammatory regulator, which in turn reduces the expression of genes associated with inflammation. NAD(P)H:quinone oxidoreductase 1 (NQO1) can also inhibit TLR-mediated innate immune responses, shielding cells from oxidative stress. NQO1 promoted IκB-ζ degradation in a ubiquitin-dependent manner through its interaction with the nuclear IκB protein IκB-ζ [30]. AHR regulates cellular metabolism by reacting to a wide range of molecules. Since the transcription factors of the bHLH/PAS family AHR and Hypoxia Inducible Factor-1 (HIF-1) signaling pathways share a dimerization partner HIF-1β (ARNT), the cellular biological behavior of ARNT was extensively studied to gain insight into the possible interactions between these signaling pathways. The production of the AHR:ARNT and HIF-1 complex can occur through dimerization between the two subunits of HIF1, HIF-1α, and HIF-1β (ARNT). The HIF-1α subunit comprising of two transactivation domains (TAD): the NH2-terminal (N-TAD) and the COOH-terminal (C-TAD), regulates aerobic metabolism and energy production to maintain oxygen homeostasis. While HIF-1β (ARNT) is widely expressed, the expression of HIF-1α is dependent on the intracellular oxygen concentration because it is ubiquitinated and is rapidly degraded by proteases in the presence of normal oxygen. Reduced oxygen availability gives HIF-1α stability, which allows it to move to the nucleus and bind ARNT, which in turn causes the expression of genes containing hypoxia response elements [31,32]. The expression of various target genes for metabolism, detoxification, and immune response, including Cytochrome P450 Family 1 Subfamily A Member 1 (CYP1A1), Cytochrome P450 Family 1 Subfamily A Member 2 (CYP1A2), and Cytochrome P450 Family 1 Subfamily B Member 1 (CYP1B1), are regulated by the AHR–ARNT heterodimer complex [33]. AHR was shown to modulate the epigenetic and nongenomic pathways in immune regulation such as the regulation of c-SRC-driven phosphorylation and the stabilization of indoleamine 2,3-dioxygenase 1 (IDO1), which is critical for the induction of endotoxin tolerance in dendritic cells (DCs) [34], microbial defense, and immunity. By controlling the T-cell subpopulation and cytokine profile in dextran sodium sulfate (DSS)-induced colitis, AHR can significantly protect against IBD. Moreover, TCDD, which functions as an AHR agonist, can lessen the symptoms of 2,4,6-trinitrobenzene sulfonic acid (TNBS)-induced colitis [35,36].

AHR was believed to be primarily involved in the metabolism of environmental chemicals, but in more recent times, researchers have become interested in AHR signaling regulation and how important it is for the best possible immune and physiological responses [37]. Hydrophobic molecules with aromatic rings, known as AHR ligands, are found in both exogenous naturally occurring substances and exogenous synthetic aromatic hydrocarbons. AHR ligands can be categorized as exogenous and endogenous. Examples of exogenous ligands include α-napthoflavone, 2-(Indol-3-ylmethyl)-3,39-diindolylmethane (Ltr-1), Indolo (3,4) bicarbazole (ICZ), 3,3-diindolylmethane (DIM), Indole-3-acetonitrile (I3ACN), flavonoids like Quercetin, Curcumin, Resveratrol, organic compounds like Benzo[a]pyrene (BaP), Benz(a)anthracene (BA), and environmental pollutant TCDD [38,39]. In addition to exogenous ligands, many endogenous ligands produced by host and microbial metabolism can act as AHR agonists (Table 1). Tryptophan metabolites play a significant role in AHR activation and AHR also regulates the rate limiting enzymes IDO, tryptophan 2,3-dioxygenase (TDO2), kynureninase (KYNU), and kynurenine 3-monooxygenase (KMO) of the TRP catabolic pathway. Dietary consumption and bacterial metabolism produce other TRP metabolites, such as indoles [37].

### 2.2. Anti-Inflammatory Role of AHR

As a component of innate immunity, the intestinal epithelial barrier is made up of intestinal epithelial cells (IECs), goblet cells, and a mucus layer, which provides an initial defense against any invasive pathogen. AHR in the epithelial barrier has a significant role in the development of IECs and goblet cells, and a lack of AHR activation results in reduced mucosal integrity, loss of gut barrier and gut bacterial translocation [45]. Previous research suggests a potential link between AHR and IBD. TCDD-induced AHR activation can alter the severity of IBD symptoms by preventing the differentiation of Th17-cells and prostaglandin E2 production [46]. Two pathways were hypothesized to explain the protective role of AHR, albeit the specific mechanism is still unknown. Firstly, AHR increases the expression of IL-22 in lymphoid cells [20]. The antimicrobial peptides lipocalin-2 and calprotectin, which preserve the balance between commensal and pathogenic bacteria, can be induced by IL-22. AHR-dependent IL-22 transcription is also triggered by microbially produced Trp metabolites, protecting the mucosa from inflammation and stopping pathogen colonization [47,48]. Secondly, AHR plays an anti-inflammatory role by controlling lymphocyte production against pathogenic microorganisms such as naturally occurring IL-22- and IL-17A-producing cells, which are highly prevalent at mucosal sites and are activated by AHR [48,49]. In TNBS mice colitis, AHR ligands NPD-0414-2 and NPD-0414-24 had a modulatory influence on the production of IFN-γ and IL-22 [50], by which AHR can maintain an anti-inflammatory effect in the gut by suppressing pro-inflammatory cytokines. Several gut bacteria and microbially produced metabolites, such as tryptamine (TrA), indole, 2-oxindole, 3-methylindole (skatole), indoxyl sulfate (IS), indole-3-acetic acid (I3A), indole-3-propionic acid (IPA), indole-3-pyruvate (IPyA), and indole-3-aldehyde, can modulate AHR response and maintain intestinal homeostasis, even though tryptophan by itself lacks the ability to bind AHR or induce AHR activity [51]. For example, the tryptophan metabolite indole-3-aldehyde from *Lactobacilli* spp. can function as an AHR ligand and is protective against pathogenic *Candida albicans* colonizing on the mucosal surface [49]. The co-evolution of commensal fungus with the immune system and microbiota facilitates the positive feedback between indoleamine 2,3-dioxygenase (IDO1) and AHR which is advantageous for host survival [34]. Gut bacteria can produce skatole from tryptophan which can breach immunological homeostasis with the development of IBD by regulating IECs apoptosis [51]. Microbiome metabolites were found to regulate AHR and its target genes in the intestine and liver. The AHR signaling pathway can, therefore, influence the microbiome’s composition. These findings imply that gut microbiome and AHR communicate in both directions [52] to play a key role in IBD pathogenesis. Research was carried out on the differences in AHR levels between healthy people and IBD patients. Research on patients with IBD has revealed variable degrees of decreased expression of AHR or no discernible differences. Patients with CD have lower levels of AHR expression than UC patients in comparison to healthy ones and this reduction is particularly noticeable in the inflammatory mucosa of CD patients [53,54].

### 2.3. T-Cell Differentiation and AHR

AHR plays a protective role against autoimmune illnesses such as diabetes [55], colitis [56], and experimental autoimmune encephalomyelitis (EAE) [57,58] by modifying adaptive immunity by controlling the T-cell response at several levels. AHR can play an anti-inflammatory role by activating CD4+ T-cells expressing CD25. These cells can suppress the production of IL-2 and limit the proliferation of effector T-cells, but they can also secrete a significant amount of IL-10 in response to antigenic stimulation [59]. FOXP3+ regulatory T- (Treg) cells secrete IL-10 to maintain immune tolerance and suppress excessive immune response and are linked to AHR through direct transactivation, control of FOXP3 transcription by expressing SMAD1 in Tregs, and modulation of DCs [57,60,61]. AHR affects the development and function of Treg-cells. CD4+ T-cells lacking AHR results in reduced accumulation of these cells in secondary lymphoid tissue [62]. AHR can cause Treg-cell differentiation to induce the expression of CD39 resulting an immunosuppressive effect and differentiation of IL-10 producing CD4+ FOXP3- T-cells through granzyme B [56,61]. In addition to its indirect effects through DCs recruitment suppressing CD8+ T-cells in both primary and secondary infection, localized production of IL-27 or its component p28 can inhibit the development of Th17-cells directly in the immune response to pathogens by inhibiting the intracellular signaling molecule STAT1 [63,64]. Long-term deficiencies in CD8+ T-cell response in primary infection upon viral challenge are also observed following AHR stimulation, which is accompanied by changes in DNA methylation and gene expression [65]. AHR activation by ligands can induce the immune system’s development: controlling gene expression and DNA methylation as T-cells react to infection later in life AHR CD4+CD8α α+ double positive intraepithelial lymphocytes (DP IELs) which display a significant role in tolerance to dietary antigens [66]. The initial immune cell to maintain tissue homeostasis at epithelial sites, gammadelta T-cells, is likewise under the control of AHR signaling. By acting as an antigen-presenting cell and secreting various cytokines and chemokines, gammadelta T-cells can affect adaptive immunity [67]. Immune homeostasis depends on the balance between Th17- and Treg-cells, and multiple autoimmune and inflammatory disorders are linked to the dysregulation of this balance. The cytokine profile and the AHR ligands can affect the intricate function of T-cell differentiation.

## 3. Gut Bacterial Changes and IBD

### 3.1. Gut Microbiome in Immune Tolerance and Homeostasis

The gut bacteria coexist in a balanced symbiotic relationship in a healthy host and can maintain immune homeostasis. Human gut microorganisms are helpful for food digestion, nutrition, and for immune system conditioning, disease prevention and maintaining gut homeostasis [68]. Disruptions in the gut microbial diversity are the root cause of many chronic diseases, such as obesity [69], metabolic syndrome [70], and multiple sclerosis [71]. Healthy individuals have a diverse and species-rich gut microbial composition. *Proteobacteria* and *Clostridium* species predominate in the human small intestine, whereas the cecum and colon are inhabited mostly by the *Bacteroidaceae* and *Clostridiaceae* families. Contrarily, mice cecum comprises of *Ruminococccae, Rikenellaceae*, and *Lachnospiraceae*, while the colon is habitat to *Bacteroidaceae, Rikenellaceae*, and *Prevotellaceae* [72]. The gut microbiota contributes to the maintenance of the intestinal epithelial barrier and influences the development and maturation of immune cells, including T-cells, B-cells, and antigen-presenting cells. Celiac disease with irritable bowel syndrome (IBS)-type symptoms is an autoimmune disease involving multiple factors, including motor dysfunction, low-grade gut inflammation, and altered gut microbiota characterized by a decrease in Gram-positive bacteria and an increase in Gram-negative bacteria. Five different strains of *Lactobacillus* and *Bifidobacterium* probiotic supplements were found to alleviate Celiac disease with irritable bowel syndrome-type symptoms in a randomized clinical trial [73]. In colitis caused by DSS, the probiotic *Saccharomyces boulardii* was shown to have anti-inflammatory activity and to change the expression of miRNA. It was also shown that gut microbiota populations in this condition upregulate the expression of inflammatory cytokines [74]. These are just a few examples of how gut bacteria can influence the production of inflammatory mediators. By damaging the mucosal layer and goblet cells, translocated gut bacteria can create biofilms, which shield microorganisms from physical stress and immune protection leading to dissemination of resistance genes and IBD progression. The primary characteristic of IBD is the adherent *Bacteroides fragilis* biofilm, which is suppressed by 5-ASA and antibiotics but not removed [75]. TLRs are potent molecular regulators that are distributed according to tissue and cell type. They are essential for the GIT’s homeostasis, development, and host defense against infections. TLRs on immune cells can recognize molecular patterns expressed by gut bacteria, such as TLR2, TLR4, and TLR5 recognize lipoteichoic acid, LPS and flagellin, respectively [76]. By affecting the synthesis of cytokines and other immune mediators, TLR activation aids in the regulation of immune responses. Less severe DSS-induced colitis, as well as fewer neutrophils and Treg-cells, were observed in TLR2 and TLR4 knockout mice [77]. Previous research on germ-free (GF) mice with compromised immune systems exhibit decreased numbers and capacity for differentiation of particular myeloid cell progenitors, potentially resulting in compromised early defenses against infections revealing the importance of gut bacteria in the development of immunological tolerance as well as decreased expression of antimicrobial peptides [78,79]. GF mice are more susceptible to microbial infections because they have lower Treg-cell counts and decreased innate lymphoid cell activity [80]. In a mutually beneficial relationship, gut commensal microbes coexist with the host and play a critical role in influencing the immune system. Various beneficial gut bacteria, such as *Bifidobacterium adolescentis* and segmented filamentous bacteria, primarily influence the modulation of gut Th17-cells [81,82]. High-fiber and fat diets can be fermented by commensal gut microflora to produce short-chain fatty acids (SCFAs) including butyrate, which can affect AHR expression and activity because they can serve as AHR ligands. GPR109A, a butyrate and niacin receptor produced by the gut microbiota, can stimulate the differentiation of Treg-cells and IL-10-producing T-cells to promote an anti-inflammatory effect in colonic macrophages and dendritic cells. However, individual variations in the composition of the gut microbiota as well as the type and length of the diet may affect the precise effects [83,84]. The convergent findings explain the contribution of intestinal bacteria to immune development and homeostasis.

### 3.2. Gut Bacterial Dysbiosis in IBD

The dysbiosis of the gut microbiome is linked to specific disease phenotypes in IBD and can be used to assess IBD disease activity, diagnosis, and prognosis [85]. Numerous studies have focused on identifying the gut microbe’s role in IBD (Table 2). Although a single animal model cannot accurately reflect the multifaceted etiology of IBD, understanding pathophysiology and creating new treatment options can be aided by genetic changes [86]. To identify the microbial community in IBD, different mouse models including chemically induced colitis (e.g., DSS and TNBS-induced colitis), genetic manipulation (e.g., spontaneous colitis in IL-10−/− and Muc2−/− mice), infectious colitis and T-cell transfer model of colitis [86,87]. Many murine models were designed to better understand the pathophysiology of IBD and to develop new treatments. Every mouse model is different and contributes to our understanding of the relationship between the gut microbiota and the epithelium barrier in the development of different immunological treatments [88]. Particularly, chemically produced models of colitis, such as the DSS-induced mice colitis model are widely studied due to their quick pro-inflammatory response by directly damaging the mucosal barrier and similarities with human UC [89]. It is also considered that using chemically induced colitis models is preferable than using immunocompromised or genetically modified mouse models [25]. The community characterization and diversity index of intestinal microbiota were linked to IBD with the growing use of next-generation sequencing techniques [90]. For example, the DSS-induced colitis model has shown an expansion of *Enterobacteriales*, Deferribacterales, Verrucomicrobiales and *Erysipelotrichales* bacteria [91]. Previous research demonstrated that lower flagellin transcripts of *Enterobacteriaceae* family in mice reduce intestinal inflammation, suggesting heterogeneous pathogenic involvement [92,93]. A 16S rRNA-based study of the T-bet−/− × Rag2−/− Ulcerative colitis (TRUC) mouse model revealed a co-relationship between colitis and the *Enterobacteriaceae* family, which includes *Proteus mirabilis* and *Klebsiella pneumoniae* [94]. IBD patients have significantly higher Proteobacteria (*Enterobacteriaceae*, *Acidaminococcus, Veillonella dispar*) than healthy controls, but Firmicutes (*Roseburia* and *Faecalibacterium prausnitzii*) and Actinobacteria proportions were lower in IBD [95]. Gram-negative bacteria, particularly pathogenic forms of *E. coli*, are a major factor in IBD. It was demonstrated that the immune system’s recognition of Gram-negative bacterial LPS using gnotobiotic TLR2−/− and TLR4−/− mice can induce ileitis [96]. Gram-positive commensal bacteria also facilitate colitis. In DSS-induced colitis mice whose Gram-positive bacteria had been eliminated using a pretreatment with vancomycin, significantly reduced recruitment of monocytes and macrophages with lower levels of pro-inflammatory cytokines were displayed, such as TNF-α and IL-6, compared to untreated or colistin-treated mice [97]. Human DC stimulation with microbial metabolite in a colitis patient raised the percentage of Th2- to Th1-cells, which exacerbated IBD with certain fecal bacteria (*Bacteroides* and *Candida)* expansions [92]. Tryptophan derivatives, which function as AHR ligands and stimulate the production of IL-22, are reduced in the gut microbiota of both IBD patients and CARD9 knock out mice [20]. Researchers have employed AHR antagonists in addition to AHR ligands to investigate the pathway that impairs AHR signaling. Using young adult mouse colonocyte (YAMC) cells, one study investigated the AHR modulation of the tryptophan microbiota metabolites. The agonist and antagonist properties of microbial tryptophan metabolites (indole-3-acetate, indole-3-aldehyde, indole, and tryptamine) were studied using three additional Ah-responsive genes, namely, CYP1A1, CYP1B1, AHR repressor (Ahrr), and 2,3,7,8- tetrachlorodibenzo-p-dioxin (TCDD)-inducible poly (ADP-ribose) polymerase (TiParp) [98]. The cytokines produced by the intestinal endothelium lymphocytes are influenced by the gut bacteria. IELs are T-cells located in the intestinal epithelium. Compared to intestinal lymphocytes from healthy controls, IELs from UC patients released significantly more IL-1β, and intestinal lymphocytes from CD patients secreted significantly more IL-17A, IFN-γ, and TNF-α [99].

## 4. Immunopathogenesis of IBD

**Figure 2:**
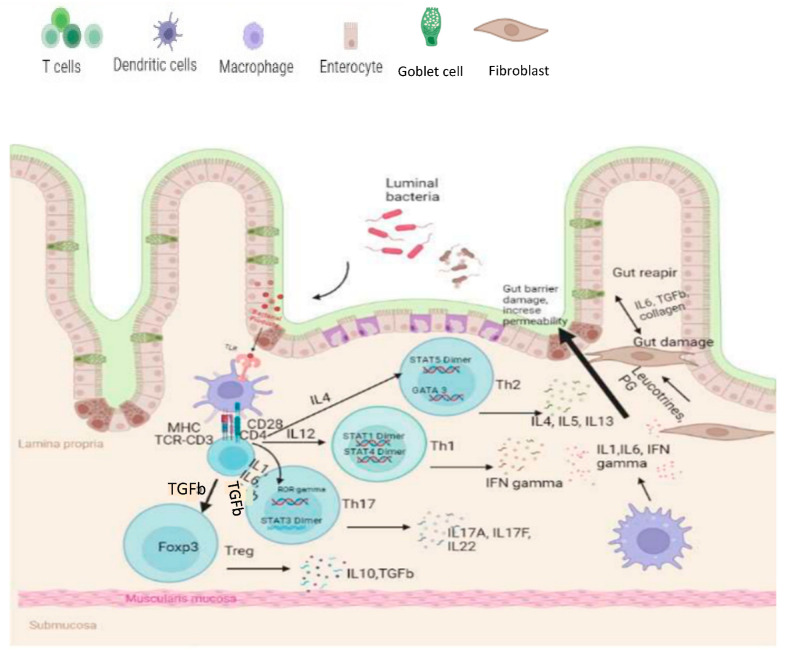
A schematic diagram of immunological basis of cellular populations and mediator changes in IBD. Bacterial dysbiosis may result in overexpression of inflammatory cytokines (IL-1β, IL-6, and IFN-γ) by dendric cells and macrophages ultimately causing increased permeability of the gut epithelial cells. While Treg-cells secrete anti-inflammatory cytokine IL-10 and fibroblasts releases collagen and TGF-β for gut tissue repair.

The largest and most intricate part of the human immune system is the intestine where a diverse population of microbes reside. These residing intestinal bacteria condition the intestinal immune system by exposing bacterial antigens to the epithelial luminal barrier and maintain immune tolerance [118] (Figure 2).

### 4.1. Epithelial Barrier Dysfunction

The epithelial lining of the gastrointestinal tract serves as a physical barrier between the luminal contents and the underlying immune system. An intact mucus layer and intestinal epithelial barrier are the defense against bacterial invasion to sustain homeostasis [69]. However, a precise balance between tolerance to self-antigens and immunological response needs to be maintained, as abnormalities in this T-cell-mediated peripheral tolerance mechanism may lead to a range of autoimmune diseases [119]. The second line of defense against bacterial invasion is the intestinal epithelium comprising enterocytes, goblet cells, and Paneth cells. The mucins secreted by goblet cells form the outer mucus layer, which is inhabitant of commensal bacteria and the inner mucus layer is sterile [120]. IECs play a role in both tolerance against commensal bacterial antigens and the immune response against exogenous pathogens. IECs maintain a tight junction and produce chemokines, cytokines, mucins, and defensins that recruit immune cells and preserve the mucosal barrier [121]. After being damaged by infectious or chemical insults, AHR had a major effect on IEC regeneration. The AHR pathway in IECs inhibited Wnt-b-catenin signaling and limited intestinal stem cell (ISC) proliferation by transcriptionally controlling *Rnf43* and *Znrf3* and E3 ubiquitin ligases that protect the stem cell niche and restore barrier homeostasis [122]. The epithelial barrier function is frequently compromised in IBD, which permits bacteria and luminal antigens to infiltrate. An immunological reaction and inflammation are brought on by this barrier break [123]. It was demonstrated that IAld ameliorates the inflammatory responses in DSS-induced colitis by reversing the inflammatory responses and reestablishing the function of the intestinal epithelial barrier, in part through AHR activation [38,124].

### 4.2. Altered Immune Response by Innate Immune Cells

The different innate immune cells (neutrophils, macrophages, DCs, and natural killer T- (NKT) cells, etc.), and innate cytokines and molecules (IL-1, TNF, and defensins) found in the intestine, lead to inflammatory response in IBD in response to gut microbial dysbiosis [125].The dysfunction of the intestinal epithelium barrier and release of various inflammatory mediators, neutrophils can prolong intestinal inflammation [5]. Intestinal macrophages are the most common type of mononuclear phagocytes capable of inducing an immune response [126]. In the inflammatory mucosa of patients with IBD, there is an increase in macrophages. In particular, macrophages expressing both DC (CD205, CD209) and macrophage (CD14, CD33, CD68) markers can initiate a rapid response to luminal microbial antigens by producing high quantities of IL-12 and IL-23 in response to microbial stimulation [127]. In intestinal homeostasis limited macrophage are produced with low proinflammatory cytokines, such as IL-1α and IL-1β, and tumor necrosis factor-alpha (TNF-α) [125]. IBD pathophysiology may include macrophage dysregulation of AHR signaling. According to some research, the AhR-Src-STAT3-IL-10 signaling pathway, which aids in tissue repair and reduces inflammation, can cause macrophage AHR activation to release cytokines like interleukin-10 (IL-10). Following LPS stimulation, AHR was highly expressed via the NF-κB pathway. IL-10 expression decreased in AHR KO macrophages, but it increased in RAW264.7-cells because of LPS-induced AHR overexpression [128]. The professional antigen presenting cell, DCs play a major role in the pathophysiology of IBD by influencing immune responses and tolerance. DCs can transport antigens from commensal microorganisms to the draining lymph nodes to interact with B- and T-cells to promote tolerance and have anti-inflammatory response by generating IL-10 [129,130]. However, in inflammatory bowel disease, low conditioning of DCs was noted because of the reduced mucosal expression of TGF-β and TSLP, downregulated retinoic acid signaling, and elevated expression of TLR2, TLR4, CD40, and chemokine receptor CCR7. All these aspects support inflammation by causing DCs to react inappropriately to captured antigens and by generating pro-inflammatory cytokines like TNF, IL-1β, IL-6, and IL-18 [1,131,132]. The NKT-cell, which generates high quantities of Th1, Th2, and Th17 cytokines to initiate an immediate immune response upon identifying phospholipids and glycolipids presented by CD1d on the antigen-presenting cells (APC), is another cell type implicated in the pathophysiology of IBD [2]. More T-cells expressing the NK marker CD161 in the inflammatory lamina propria of Ulcerative colitis patients produce more IL-13 in response to CD1d [133].

### 4.3. T-Cell Response in IBD

The inflammatory response over time is maintained by commensal bacteria to activate the adaptative immune response mediated by effector CD4+ T helper cells with the synthesis and release of several proinflammatory mediators, such as reactive oxygen and nitrogen metabolites, eicosanoids, chemokines, and cytokines, which are enhanced in the intestine [134]. Effector CD4+ T-cells frequently display a unique cytokine profile and functional heterogeneity, which are related to the kind of antigenic stimulation. Treg-cells were discovered to be essential for the prevention of autoimmune colitis [135]; however, the precise mechanism by which Treg-cells maintain immunological tolerance is still unclear [136]. Multiple aspects of molecular and cellular events are involved in Treg suppressive immune response. One of the possible mechanisms is that Treg inhibits Th17-cell growth and differentiation [137]. Th17-cells are a subpopulation of effector T-cells that originate from naïve CD4+ T-cells in response to TLR3, TLR4, or TLR9 in the presence of IL-6 and TGF-β [138]. T-cell development occurs in the thymus but differentiation of Th-cells into subpopulations occurs after an encounter with an antigen. The interactions of cytokines with STAT proteins play a key role in the differentiation of T-cell types. When certain cytokines are present, APCs prepare and deliver an antigen to naive T-cells by identifying microbe-associated molecular patterns (MAMPs), causing them to differentiate into Th1-, Th2-, Treg-, and Th17-cells. Each committed cell contributes to the growth of many immune-related human disorders in addition to their defense against invasive infections. As shown by the prevalence of cytokines associated with Th1-cells in the mucosa, such as IL-12 and IFN-γ, human CD was linked to Th1-cells, whereas UC is characterized by an enhanced production of IL-5 and IL-13, leading to its relationship with Th2-cells [139,140]. Th17-cells that may produce IL-17A have a significant pathogenic role in chronic inflammatory diseases like rheumatoid arthritis by osteoclastic bone resorption and IL-17 mRNA is detectable in biopsies from cutaneous inflammation (lesion from psoriatic skin) [141,142,143]. In murine models, TGF-β drives the differentiation of naive T-cells to Treg-cells, whereas Th17-cells are induced in the presence of IL-6 and TGF-β [144,145]. The cytokine microenvironment for the development of Th17-cells in humans is more complex suggesting that IL-1β, IL-6, TGF-β, IL-21, and IL-23 are all necessary for the differentiation of human Th17-cells [146]. When monocytes and dendritic cells are stimulated by LPS and peptidoglycan, they release a lot of IL-1β and IL-6 but not much IL-12, which leads to the development of naïve T-cells into Th17 [147]. The cytokines IL-6 and IL-23 produced by APC stimulate the JAK-STAT pathway, which is crucial for Th17 differentiation [144,148]. STAT3 can regulate the expression of the transcription factor RORᵧ, which is essential for the development of immunological tolerance, suppression of RORᵧ expression results increased expression of TGF-β and FOXP3 which encourages the development of T-cell subpopulations other than Th17-cells [146]. Some of the cytokine receptors expressed by Th17-cells include the IL-6 receptor (IL-6R), the transforming growth factor-beta (TGF-β) receptor, the IL-23 receptor, the IL-21 receptor, and the IL-1 receptor. The presence of IL-6, IL-1β, and IL-23 at high concentrations induces Th17-cell differentiation and results in gut inflammation [144,146]. TNF-α and IL-1β support the development and differentiation, while IL-6 is regarded as a necessary cytokine for Th17 differentiation. It appears that IL-23 is a key proinflammatory cytokine that activates Jak2, PI3K/Akt, STAT3, and NFκB to stimulate the production of IL-17 in CD4+ T-cells [149,150]. While activated DC secretes IL-23, which was suggested to be essential for Th17 differentiation, its precise role in a secondary immune response remains unclear. However, IL-23 can induce an activation state that differs from the well-studied Th1 and Th2 profiles [151]. Even though Th17-cells secrete IL-17A and draw neutrophils and macrophages to the site of injury to eliminate extracellular infections, the balance between Treg and Th17-cells is crucial to maintain immunological homeostasis. The pathogenesis of IBD is influenced by the increased production of the key effector cytokine IL-17A released by Th17-cells. IL-17A and IL-17F both activate several signaling pathways related to downstream innate immunity receptors. Thus, Th17-cells can enhance the inflammatory processes in the gut by promoting the activation of both innate and adaptive immune response mechanisms [152]. Th17-cells can differentiate into Th1-cells, a process that may be important in the onset and progression of inflammatory disorders [153], explaining how environmental stimuli influence Th17 functional plasticity. Th17-cells enhance intestinal homeostasis by IgA synthesis and IL-22 production. Administration of FICZ, a ligand of AHR can increase IL-22 expression that prevents and even heals colitis [53].

## 5. Conclusions

AHR maintains intestinal immune homeostasis through a variety of gut cell types, including ILCs, IELs, and IECs. AHR ligands also have a variety of sources (diet, environment, and gut microbial metabolites), structures, and modes of action. Insufficient or excessive AHR activation can lead to altered immune response and intestinal immune disorders. It was demonstrated that a few AHR agonists, including TCDD and FICZ, reduce colitis symptoms.

In conclusion, considering the beneficial effects of certain bacteria in gut homeostasis and the developments in our knowledge of the pathophysiology of IBD, AHR-based treatments emphasizing prebiotics or metabolites made from probiotic bacteria may offer a novel alternative treatment for IBD, owing to the presence of altered AHR signaling with the gut bacterial dysbiosis in IBD patients. However, the relationship between AHR and IBD is complex and not fully understood and more research is needed to prove the safety and efficacy of AHR-related treatment.

## Figures and Tables

**Table 1:** List of AHR endogenous ligands.

Ligand	Source	Class	Ref.
Indole acrylic acid (IA) Indole-3-acetic acid (IAA) Indole-3-propionic acid (IPA) Indole-3-lactic acid (ILA) Indole-3-aldehyde (IAld) Indole-3-acetaldehyde (IAAld)	Diet	Agonist	[38]
L-Kynurenine (Kyn) Kynurenic acid (KA) 6-Formyl indolo (3,2-b) carbazole (FICZ) Indoxyl-3-sulfate (I3S) Tryptamine Tryptanthrin Xanthurenic acid	Tryptophan catabolic pathway	Agonist	[39,40,41,42]
Bilirubin Biliverdin Lipoxin A4	Host metabolism	Agonist	[43,44]

**Table 2:** Altered gut bacteria in IBD in comparison to healthy host.

IBD Subtype	Gut Bacteria	Abundance Compared to Healthy	Possible Immunological Function	Ref.
UC, CD	*Faecalibacterium*	Low	Low SCFA, anti-inflammatory cytokine	[95,100]
UC	*Prevotella*	High	Mucin-degrading bacteria, increase CCL5	[101,102]
UC, CD	*Clostridium* IXa and IV groups	Low	Impaired mucin secretion and altered epithelial barrier	[103]
UC, CD	*Enterococcus*	High	Increase IL-12, IFN-γ	[104]
UC, CD	*Lactobacillus*	Low	Enhance FOXP3 expression, IFN-γ producing T-cells and increase intestinal IL-22 production, low SCFA, increase in Treg-cells	[99,105]
UC, CD	*E. coli*	High	Increase IL-12, IFN-γ	[103,106]
UC, CD	*Bacteroides*	Low	Stimulate Treg-cells to produce anti-inflammatory cytokine IL-10, immunological tolerance	[81,105]
UC	*Fusobacterium*	High	Enhanced mucosal attachment with invasion	[107,108]
UC, CD	*Campylobacter jejuni*	High	Potentially activates harmful genes of *Escherichia coli*, damaging the epithelial barrier and increasing the production of pro-inflammatory cytokines	[109]
UC, CD	*Clostridium difficile*	High	Gut bacterial dysbiosis	[110]
UC	*Desulfovibrio*	High	Increase inflammation and epithelial cell damage	[111,112]
UC, CD	*Bilophila*	High	Gut bacterial dysbiosis	[111]
UC, CD	*Ruminococcus*	High	Increase inflammatory cytokine secretion by innate immune cells	[113,114]
UC, CD	*Streptococcus*	High	Pro-inflammatory response through TLR2	[115]
UC, CD	*Akkermansia*	Low	Anti-inflammatory role by controlling the growth of total mucosa associated bacteria	[116]
UC, CD	*Bifidobacterium*	Low	Low SCFA, anti-inflammatory cytokine	[81,117]
